# An Excellent Lecture and Demonstration

**Published:** 1920-08-14

**Authors:** 


### DISEASES OF THE INTERNAL EAR.

### An Excellent Lecture and Demonstration.

At the Central London Nose, Throat, and Ear Hospital a number of otologists met recently to attend a lantern demonstration illustrating "Diseases of the Internal Ear," by Dr. J. S. Fraser, of Edinburgh. We may indeed congratulate the lecturer upon the work that he has done.

### Head Injuries.

After showing a series of slides illustrating fractures of the base of the skull, and demonstrating the relative importance of those which ran along the length of the tegmen tympani and of those which crossed the petrous portion of the temporal bone, Dr. Fraser threw on the screen pictures from slides obtained from patients who had died as the result of injuries received during the war. The first was from a man who had been struck in the parietal region without the missile having caused any direct injury to the auditory apparatus; the other two were from patients who had been admitted to a casualty clearing station suffering from severe shock as the result of multiple wounds from a high-explosive shell without any wound of the cranium. In all three hæmorrhage around the nerve in the internal auditory meatus was found.

### Labyrinthitis of Otitic Origin.

From this he passed on to that labyrinthitis which begins in the middle ear and passes on to meningitis. He reminded his audience how this may arise clinically either in the course of an acute otitis media or as the result of old chronic diseases, and said that in the former the means of access to the internal ear is through one of the windows, and in the latter from caries of the bone surrounding the lateral semicircular canal. He then showed slides from a striking case of each of these forms. The first was from a patient who had died of meningitis secondary to an acute otitis which had not caused a perforation of the tympanic membrane. On the one side the slide showed a suppurative labyrinthitis, which had spread to the meninges, and a middle ear choked with pus and exudate of an acute inflammation. The drum-head was still intact, but in its substance was a minute abscess on the point of bursting; the fibrous layer had already given way, and pus had formed in the sub-cuticular region. As the lecturer said, had this patient lived but a few more hours this drum-head would have burst and the discharge from the ear have become established. On the other side an earlier stage was seen. The middle ear again was filled with pus, the drum-head quite intact, and suppurative labyrinthitis had not yet occurred; but in the labyrinth hæmorrhages already had occurred indicative of an early stage of inflammation. Dr. Fraser compared this to the blood-stained serum which so often exudes through the hole made on paracentesis in early cases of acute otitis. The other case was one of chronic otitis media, in which tonsils and adenoids had been removed and a polypoid mass of the ear had been curetted. The patient died from meningitis, and when sections of the ear were made there was found a pan-labyrinthitis which had evidently long been there. We do not suppose that such cases are common, but that such are possible must be borne in mind whenever an operation is performed upon a case of chronic otitis media, however trivial the manipulations may appear to be.

### Labyrinthitis of Meningitic Origin.

Dr. Fraser is of opinion that all cases of labyrinthitis other than those of otitic origin spread to the internal ear from the meninges rather than by the blood-stream, and showed slides from two cases in support of this view. One was from a patient with cerebro-spinal meningitis, and the other from one with measles in whom meningitis supervened and in whom the middle ears were not affected by suppuration. He suggests that in cases of syphilis in which the internal ear is involved a similar spread has occurred. Such a view emphasises the necessity to think seriously of cases of lues in which there is any degree of internal nerve deafness.

We have no space fully to epitomise that part of Dr. Fraser's demonstration which dealt with oto-sclerosis, with congenital syphilis, with tuberculosis, and with Menière's disease, but we congratulate the Central London Nose, Throat, and Ear Hospital upon the success of the demonstration. There is far too little professional intercourse between the London Schools of Medicine and those of the provinces and of Scotland and Ireland.

